# Engineering Ru@Pt Core-Shell Catalysts for Enhanced Electrochemical Oxygen Reduction Mass Activity and Stability

**DOI:** 10.3390/nano8010038

**Published:** 2018-01-12

**Authors:** Ariel Jackson, Alaina Strickler, Drew Higgins, Thomas Francisco Jaramillo

**Affiliations:** 1 Department of Chemical Engineering, Stanford University, Stanford, CA 94305, USA; arieljackson@gmail.com (A.J.); astrick@stanford.edu (A.S.); dhiggs@stanford.edu (D.H.); 2 SUNCAT Center for Interface Science and Catalysis, SLAC National Accelerator Laboratory, Menlo Park, CA 94025, USA

**Keywords:** core-shell nanoparticles, electrocatalysis, oxygen reduction reaction, fuel cells, electrochemical energy technologies, nanomaterials synthesis

## Abstract

Improving the performance of oxygen reduction reaction (ORR) electrocatalysts is essential for the commercial efficacy of many renewable energy technologies, including low temperature polymer electrolyte fuel cells (PEFCs). Herein, we report highly active and stable carbon-supported Ru@Pt core-shell nanoparticles (Ru@Pt/C) prepared by a wet chemical synthesis technique. Through rotating disc electrode testing, the Ru@Pt/C achieves an ORR Pt mass-based activity of 0.50 A mg_Pt_^−1^ at 0.9 V versus the reversible hydrogen electrode (RHE), which exceeds the activity of the state-of-the-art commercial Pt/C catalyst as well as the Department of Energy 2020 PEFC electrocatalyst activity targets for transportation applications. The impact of various synthetic parameters, including Pt to Ru ratios and catalyst pretreatments (i.e., annealing) are thoroughly explored. Pt-based mass activity of all prepared Ru@Pt/C catalysts was found to exceed 0.4 mg_Pt_^−1^ across the range of compositions investigated, with the maximum activity catalyst having a Ru:Pt ratio of 1:1. This optimized composition of Ru@Pt/C catalyst demonstrated remarkable stability after 30,000 accelerated durability cycles (0.6 to 1.0 V vs. RHE at 125 mV s^−1^), maintaining 85% of its initial mass activity. Scanning transmission electron microscopy energy dispersive spectroscopy (STEM-EDS) analysis at various stages of electrochemical testing demonstrated that the Pt shell can provide sufficient protection against the dissolution of the otherwise unstable Ru core.

## 1. Introduction

A critical challenge for human society is to replace energy produced by fossil fuel consumption with renewable sources, such as wind and solar. The large-scale implementation of these renewables into the energy sector requires that their intermittency is addressed by developing reliable energy storage techniques. One promising storage mechanism is in the form of chemical bonds, whereby wind and solar energy can be used to synthesize energy-dense chemical fuels [1,2,3], which can then be stored or transported as needed. In the case of the transportation sector, which accounts for roughly 29% of global energy consumption [4], hydrogen is an ideal energy carrier that can be synthesized electrolytically by coupling to wind or solar sources [5]. Furthermore, hydrogen is an energy dense molecule that, when consumed in a low temperature polymer electrolyte fuel cell (PEFC) can enable driving ranges over 300 miles for a midsize vehicle [6], provide fast refueling times that are comparable with gasoline [7] and enable energy conversion efficiencies unrivalled by conventional internal combustion engines [8].

The successful large-scale commercialization of PEFCs is still hindered by cost and durability challenges [9]. At the source of both of these issues is the lack of high performance, inexpensive catalysts that are operationally robust for the kinetically-limited oxygen reduction reaction (ORR) at the fuel cell cathode [9,10,11]. Currently, Pt is the best-known mono-metallic ORR catalyst in acidic media. The extensive reliance on Pt catalysts, however, prohibits the techno-economically viable production of PEFCs at scale due to the high cost, limited natural abundance and insufficient performance of this noble metal [10]. In order to reduce the Pt loading and achieve fuel cell system cost savings, ORR catalyst performance must be enhanced in two critical areas: Pt mass-based activity and operational stability.

With respect to ORR electrocatalysis, it is well known that Pt surfaces overbind oxygen [12]. By slightly weakening the oxygen binding energy of Pt surfaces, ORR activity improvements have been demonstrated [12,13]. This is commonly accomplished by alloying Pt with transition metals, such as Co [14,15,16,17,18,19], Ni [20,21,22,23,24,25], Fe [26,27,28] and Cu [29,30,31]. This improvement in activity generally comes at the expense of catalyst durability due to the instability of these transition metals under the acidic conditions encountered during fuel cell operation [32,33]. Careful morphological control and annealing processes [22,34], or the incorporation of additional transition metal dopants (i.e., Mo into a Pt_3_Ni alloy) [35] have been demonstrated experimentally to improve catalyst stability. Alternatively, stability improvements can be gained by identifying transition metals with very negative heats of alloy formation with Pt, so as to kinetically inhibit transition metal diffusion and dissolution [36]. This computational descriptor was initially used to identify Pt-Y alloys as highly active and durable ORR catalysts [37] and was later extended to other Pt-lanthanide alloy systems [38,39,40,41]. In these catalysts, the active phase comprises a Pt overlayer formed during electrochemistry in acidic electrolyte that is compressively strained versus bulk Pt by the underlying Pt-lanthanide substrate. The nanoparticulate analog to this configuration is core@shell nanoparticles (with the composition metal@Pt). These high surface area nanostructures are more amenable to device integration and have provided a practical route to achieve ORR activity enhancements [42,43,44,45,46,47,48,49].

Recently, the Ru@Pt system has demonstrated enhanced ORR activity over pure Pt in both single crystals [50] and core-shell nanoparticles [51,52]. In our previous work, we developed carbon supported Ru@Pt nanoparticles (Ru@Pt/C) using a liquid phase synthesis approach [52]. While these Ru@Pt/C nanoparticles provided a significant improvement in specific (on a Pt surface area basis) activity, this enhancement did not translate to an increase in Pt mass-based activity, likely due to the relatively large nanoparticle size and Pt shell thicknesses. Furthermore, the electrochemical stability of these Ru@Pt/C was not extensively explored.

Herein, we systematically explore the preparation of Ru@Pt/C nanoparticles and are able to achieve a major enhancement in Pt-based mass activity over our previous studies. This was accomplished by leveraging the high specific activity of Ru@Pt/C and by tuning synthesis parameters to minimize the amount of subsurface Pt. With thinner Pt-shells and reduced average nanoparticle diameters, the mass activity of the Ru@Pt/C catalyst demonstrates activity of 0.50 A mg_Pt_^−1^ at 0.9 V vs. RHE, exceeding the 2020 DOE mass activity target (0.44 A mg_Pt_^−1^) [53]. The electrochemical stability of Ru@Pt/C was also investigated by an accelerated durability testing protocol that involved 30,000 electrochemical cycles from 0.6 to 1.0 V vs. RHE (125 mV s^−1^). Ru@Pt/C nanoparticles notably demonstrated remarkable stability, maintaining 85% of their initial Pt-based mass activity after this testing protocol. Due to the high electrochemical activity and stability of Ru@Pt/C core-shell nanoparticles towards the ORR, these materials can be considered promising for PEFC applications, with the next step involving performance evaluation in a lab-scale membrane electrode assembly (MEA).

## 2. Results & Discussion

### 2.1. Preparation and Characterization of the Ru@Pt/C Catalyst

**Figure 1:**
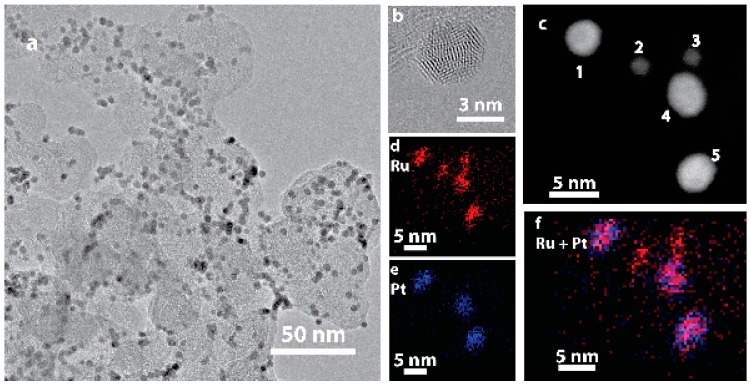
TEM imaging of (**a**) as-prepared Ru@Pt/C; (**b**) HRTEM image of a single supported Ru@Pt/C nanoparticle; (**c**) ADF-STEM image of five catalyst particles, numbered 1–5, exhibiting two distinct sizes; (**d**–**f**) EDS maps of Ru (red) and Pt (blue). The two small particles are uncoated Ru particles, while the three large particles contain both Ru and Pt.

Ru@Pt/C nanoparticles with a Ru:Pt molar ratio of 1:1 were synthesized following a previously reported two-step, one-pot polyol procedure [52,54] and then loaded onto a high surface area carbon (Vulcan XC-72) support. A transmission electron microscopy (TEM) image of the as-prepared catalyst is shown in Figure 1a. TEM determined particle sizes are tabulated in Table S1. High resolution TEM imaging (Figure 1b) demonstrated that these particles are crystalline as indicated by their lattice fringes. An annular dark field scanning transmission electron microscopy (ADF-STEM) image of five particles is shown in Figure 1c, along with elemental maps obtained by STEM energy dispersive spectroscopy (STEM-EDS) imaging provided for Ru (red, Figure 1d), Pt (blue, Figure 1e) and combined Ru/Pt (magenta, Figure 1f). Two distinct particles sizes are observed. The larger particles (labeled 1, 4 and 5 in Figure 1c) contain both Pt and Ru. Although the resolution of the EDS map is limited by signal intensity and sample drift, the combined Ru/Pt map indicates that the Ru is concentrated in the center of the particle while Pt is concentrated on the particle edge, consistent with a Ru@Pt/C core-shell structure. The smaller particles (labeled 2 and 3) contain only Ru. There was no evidence of any as synthesized Pt-only particles. The bimodal nanoparticle size distribution (Figure S1) is a consequence of incomplete coating of some of the Ru core particles. Although further synthesis optimization could increase the yield of coated particles, the presence of Ru-only cores should not have an appreciable effect on electrochemical performance, as electrochemical conditioning removes uncoated Ru particles [55].

### 2.2. Electrochemical Characterization and Activity

**Figure 2:**
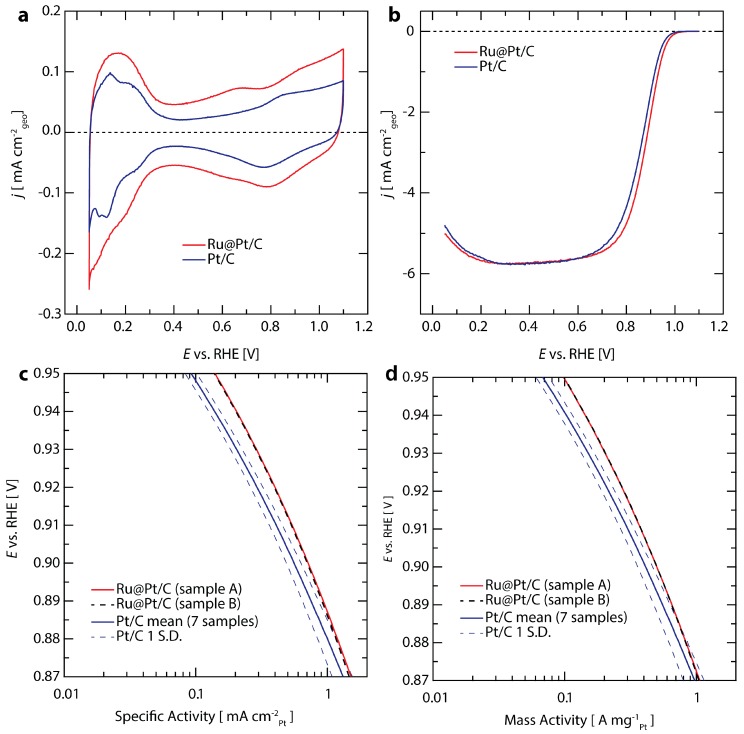
Electrochemical characterization comparing Ru@Pt/C (red) and Pt/C (blue). All tests were performed at 20 mV s^−1^ in 0.1 M HClO_4_ at 1600 rpm using a reversible hydrogen electrode. (**a**) Cyclic voltammograms in N_2_ saturated electrolyte; (**b**) Anodic direction linear sweep voltammograms in O_2_ saturated electrolyte; Pt-based (**c**) specific and (**d**) mass activity of the catalysts. The two sister samples of Ru@Pt/C (shown in red and dashed-black lines) demonstrate nearly identical performance. Seven samples were tested for Pt/C, with the average activity shown in blue and one standard deviation (S.D.) in each direction shown with dashed-blue lines.

The electrochemical properties and performance of Ru@Pt/C catalysts in 0.1 M HClO_4_ are shown in Figure 2, alongside commercial Pt/C (46.6 wt % Pt from TKK) for comparison. Baseline cyclic voltammograms of Ru@Pt/C and Pt/C conducted in N_2_ saturated electrolyte between 0.05–1.1 V are shown in Figure 2a. The loading for each sample is between 6–6.3 µg_Pt_ cm^−2^. The hydrogen underpotential adsorption/desorption and OH*/O* adsorbate redox features that are typical for exposed Pt surfaces in aqueous electrolytes are observed between 0.05–0.4 V and above ~0.7 V vs. RHE, respectively [56]. Using the charge transferred in the H_upd_ region, similar electrochemically accessible surface areas (ECSA) of 70 m^2^ g_Pt_^−1^ for Ru@Pt/C and 72 m^2^ g_Pt_^−1^ for commercial Pt/C were determined. The higher double layer capacitance observed for Ru@Pt/C is due to its higher carbon content (20 wt % Ru/Pt on C for Ru@Pt/C versus 46.6 wt % Pt on C for Pt/C) while its similar Pt loading and Pt-based ECSA result in similar capacitance subtracted H_UPD_ area. None of the characteristic Ru peaks are observed [57,58], indicating that the Ru cores are completely coated [59] and the uncoated Ru cores present after synthesis have been successfully removed with the post synthesis electrochemical conditioning [55]. The electrochemical potential ranges of the H_UPD_ and OH*/O* regions on the Ru@Pt/C catalyst are shifted compared with Pt/C (see Figure S2 and Discussion in Section 4.1 in the Supplementary Materials). This is consistent with previous results [60] where atomic layers of Pt on single crystal Ru were shown to shift the H_UPD_ region cathodically and the OH*/O* region anodically by roughly the same magnitude. We observed a shift of ~24 mV for H_UPD_ and OH*/O* (see Figure S2).

The ORR activity was evaluated using an anodic linear potential sweep in O_2_ saturated 0.1 M HClO_4_ between 0.05–1.1 V vs. RHE and applying background capacitance and electrolyte resistance corrections (Figure 2b). Both Pt/C and Ru@Pt/C achieve the theoretical mass-transport limited geometric current density for a 4-electron reduction process of ca. 5.7 mA cm^−2^ at potentials lower than ~0.7 V vs. RHE [61]. In the potential range between 0.8 and 1.0 V vs. RHE, the measured current reflects a mixture of kinetic and mass transport effects. Kinetically limited current densities were determined using the Koutecky-Levich correction (see Supplementary Materials for details) and are plotted on a specific activity basis using Pt surface areas determined by H_UPD_ in Figure 2c and on a Pt mass basis normalized using inductively coupled plasma mass spectroscopy (ICP-MS) in Figure 2d. The average activity and standard deviation of seven Pt/C samples (depicted by the solid and dashed blues lines, respectively) is compared to two equivalent Ru@Pt/C sister samples (shown in red and black dashed lines) in Figure 2c. At 0.9 V vs. RHE, the average specific activity of Pt/C is 0.55 mA cm_Pt_^−2^, in agreement with the upper end of literature reported values for electrodes prepared under similar conditions [62,63]. The average specific activity of Ru@Pt/C is 0.70 mA cm_Pt_^−2^, representing a 27% improvement over that of state of the art commercial Pt/C likely resulting from ligand and strain effects induced by Ru core [52,64,65].

At 0.9 V vs. RHE, the mass activity of commercial Pt/C is 0.41 A mg_Pt_^−1^ (Figure 2d), once again in line with previously reported literature values [62,63]. The mass activity of Ru@Pt/C is 0.50 A mg_Pt_^−1^, exceeding the DOE 2020 target [53] of 0.44 A mg_Pt_^−1^ and representing a 22% improvement over Pt/C. We also compared the Ru@Pt/C with a Pt catalyst prepared in house using the same synthesis technique and treatments as those used for the Ru@Pt/C (replacing the Ru precursor with a Pt precursor). The Ru@Pt/C provided a 2.8-fold greater mass activity than the in-house prepared Pt sample (see Supplementary Materials and Figure S3 for more details).

Although Ru is approximately one twentieth the cost of Pt, it is still a precious metal with a 5-year average price of 2 $/g, compared to 40 $/g for Pt (August 2012–August 2017) [66]. Ru makes up 33% of the mass of the Ru@Pt/C catalyst based on nominal precursor loading, which results in a total platinum group metal (PGM) activity of 0.33 A mg_PGM_^−1^ and a 20% reduction in catalyst cost (0.08 $/A versus 0.10 $/A for Ru@Pt/C versus Pt/C, respectively). This is a conservative estimate for the Ru@Pt/C system, since the synthesis resulted in a significant amount of “inactive Ru” (uncoated cores) that is removed during catalyst conditioning and not actually present during ORR activity measurements. For this reason, the true total metal activity of Ru@Pt/C core-shell particles is likely higher than the value calculated here. Improvements in the synthesis uniformity and yield of coated core-shell Ru@Pt/C particles, would reduce the fraction of “inactive Ru” and therefore improve the total metal activity.

With similar ECSA values determined by H_UPD_, the increase in specific surface activity of Ru@Pt/C culminated in an increased Pt mass-based activity versus commercial Pt/C. This is in contrast to our previous report on Ru@Pt/C catalysts, where a large increase in specific activity did not translate to an increase in mass activity [52]. The catalysts in the previous report (designated as “Ru@Pt-a/C”) differed from the catalysts in this study in two major ways: (i) a higher ratio of Pt to Ru precursor was used in the synthesis, resulting in thicker Pt-shells; and (ii) the previous catalysts underwent an annealing process at 300 °C in H_2_ for 2 h that resulted in particle coarsening (Figure S4). By significantly reducing the Pt shell thickness and avoiding particle coarsening, we take advantage of the increased per site activity of the Ru@Pt/C system to improve the technologically relevant mass activity. Compared to our previous Ru@Pt-a/C report, the catalyst presented in this work has a greater than 7-fold improvement in mass activity.

### 2.3. TEM Characterization after Electrochemical Testing

**Figure 3:**
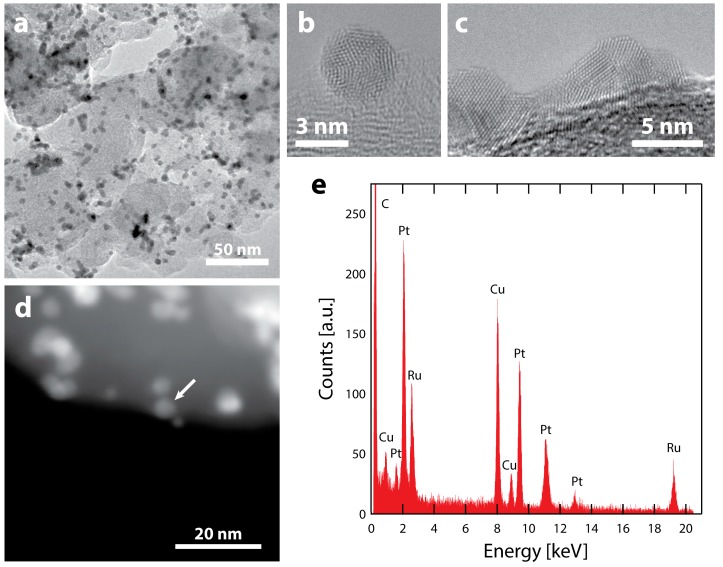
TEM characterization of Ru@Pt/C after electrochemistry (consisting of conditioning up to 1.55 V vs. RHE, cleaning and cycling in N_2_ and O_2_ up to 1.1 V vs. RHE) (**a**) Supported Ru@Pt/C catalyst particles. The electrochemical treatment results in some particle coalescence but there are also intact particles. HRTEM images are shown of (**b**) an intact Ru@Pt/C particle and (**c**) a larger particle likely coalesced from 2 or more primary Ru@Pt/C particles. (**d**) ADF-STEM image of Ru@Pt/C particles on their carbon support. (**e**) An EDS spectrum of the particle designated with the arrow. The spectrum shows a clear signal for the Ru that remains after electrochemical treatment and testing. As expected, there is also a strong signal from Pt (as well as Cu from the TEM grid and C from the support and TEM grid). The spectrum is representative of several particles that were analyzed.

TEM images and analysis after electrochemical testing (Figure 3a) show that the majority of nanoparticles remain intact after electrochemistry, with their initial spherical morphology (Figure 3b). However, a portion of the particles coarsened through coalescence, forming larger agglomerates with non-spherical morphologies (Figure 3c). Despite the particle coarsening, the high specific surface area of 70 m^2^ g_Pt_^−1^ demonstrates that coarsening is much less severe than our previous reports of Ru@Pt/C catalysts that included a H_2_ annealing process (Ru@Pt-a/C) [52,55]. This is clearly seen by the differences in particle sizes. Many of the agglomerates in our previous Ru@Pt-a/C catalysts were hundreds of nanometers in size (Figure S4), while the catalysts studied in this report are limited to tens of nanometers. Most of the Ru@Pt/C particles that do remain intact are spatially separated, indicating that further improvements in particle dispersion could reduce coarsening and thus improve surface area and mass activity.

A dark-field STEM image of the catalyst after electrochemistry is shown in Figure 3d and EDS spectra were obtained at various locations (Figure 3e, Figures S5 and S6) to examine how electrochemical testing affects particle compositions. In all cases, both Pt and Ru are observed in the various nanoparticles. As Ru is known to be unstable in acid under oxidative conditions [67,68,69], the presence of a Ru signal indicates that the Pt shell protects Ru even after exposure to potentials as high as 1.55 V vs. RHE. As previously observed [55], Ru will remain in the core of protected core-shell particles. However, in that demonstration, the catalyst was annealed at elevated temperature (300 °C in H_2_), which has been shown to improve stability and resistance to dissolution [70]. The fact that a Ru signal is detected in Figure 3e, even without any annealing treatment, indicates that the Pt shell offers protection of the Ru core under these very harsh electrochemical conditions. This is supported by the fact that no particles composed of only Ru were observed, as they were most likely removed during electrochemistry in the absence of a protective Pt shell structure.

### 2.4. Sensitivity to Pt:Ru Ratio

**Figure 4:**
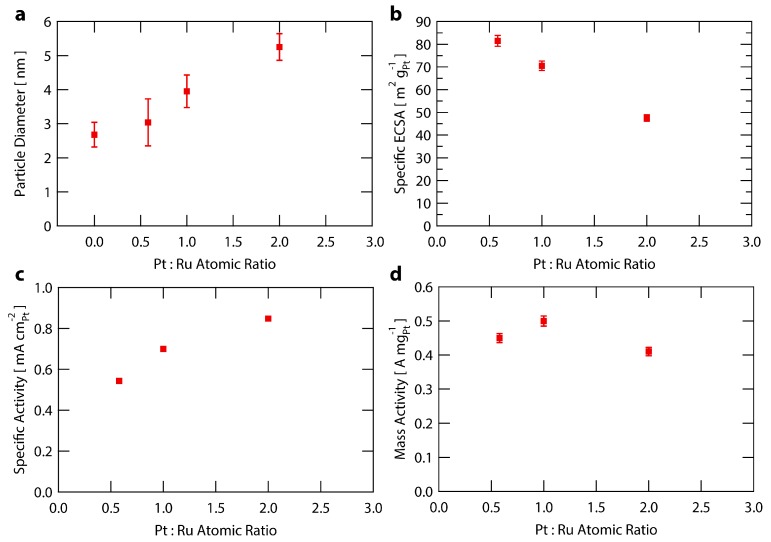
Analysis of Ru@Pt/C catalysts as a function of Pt:Ru ratio used in the synthesis. (**a**) Particle size of Ru@Pt/C catalysts as measured by TEM. Error bars represent one standard deviation; (**b**) Specific ECSA (surface area) of Ru@Pt/C catalysts. The error bars are one standard deviation associated with the average mass loading (see Supplementary Materials for further details); (**c**) Specific activity of Ru@Pt/C catalysts at 0.9 V vs. RHE. The error bars for one standard deviation are smaller than the data points; (**d**) Mass activity of Ru@Pt/C catalysts at 0.9 V vs. RHE. The error bars are one standard deviation associated with the average mass loading (see Supplementary Materials for further details).

To examine the atomic composition sensitivity of the Ru@Pt/C system, three syntheses were performed with different amounts of Pt while holding the Ru content constant. The molar ratios of Pt:Ru used were 0.6, 1.0 and 2.0 (with samples denoted Ru@Pt_0.6_/C, Ru@Pt_1_/C and Ru@Pt_2_/C). Each sample of Ru@Pt/C was exposed to the same post synthesis treatment conditions as described earlier and in the Supplementary Materials. The samples were analyzed for particle size distribution, specific ECSA, specific activity and mass activity. The results are summarized in Figure 4 and Table S1.

Figure 4a shows an increase in particle size with higher amounts of Pt added to the synthesis (increased Pt:Ru ratios), indicating an increase in the average Pt shell thickness. This is corroborated by the relationship between specific ECSA and Pt:Ru ratio shown in Figure 4b, whereby the ECSA decreases as more Pt is added to the synthesis. This trend of lower ECSAs at higher platinum contents is accompanied by an increase in specific activity (Figure 4c), likely arising from larger nanoparticles having a decreased proportion of under coordinated Pt surface sites that are not ideal for ORR owing to their increased oxygen binding energies [42,62,71]. This leads to a delicate tradeoff between specific activity and mass activity, as the overall higher coordinated surfaces associated with increased particle sizes come at the expense of higher amounts of Pt remaining inaccessible within the bulk of the nanoparticles. The result is that Ru@Pt_1_/C provides optimal mass activity (Figure 4d) over that of Ru@Pt_0.6_/C or Ru@Pt_2_/C because it more optimally balances the trends in specific activity and Pt surface area. Throughout the range of compositions tested, the Ru@Pt/C samples all exhibit a mass activity >0.4 A mg_Pt_^−1^, regardless of the amount of Pt added during synthesis. This indicates the robustness of the Ru@Pt/C system with respect to variations in this synthetic parameter.

### 2.5. Electrochemical Stability

**Figure 5:**
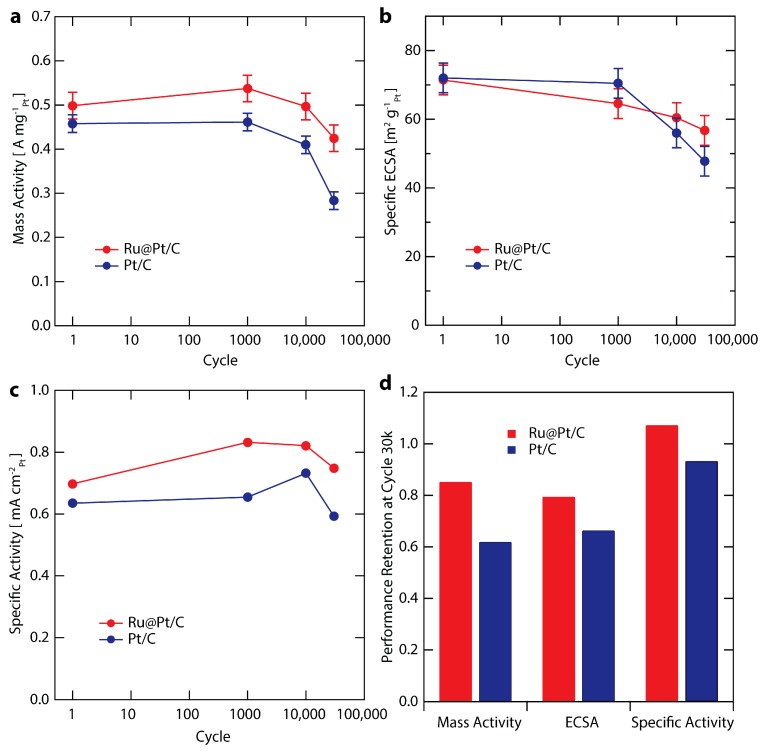
Stability test comparing Ru@Pt/C (red) and Pt/C (blue) at cycles 1, 1000, 10,000 and 30,000. The stability test involves cycling in O_2_ saturated 0.1 M HClO_4_ from 0.6–1.1 V vs. RHE at 125 mV s^−1^ at room temperature. The error bars are two standard deviations associated with the average mass loading (see Supplementary Materials for further details). (**a**) Mass activity at 0.9 V vs. RHE; (**b**) Specific ECSA (surface area normalized to mass); (**c**) Specific Activity at 0.9 V vs. RHE; (**d**) Retention of activity and surface area at cycle 30,000 relative to cycle 1.

To explore catalyst stability, we investigated the performance of the highest mass activity Ru@Pt/C sample, Ru@Pt_1_/C (referred to simply as Ru@Pt/C throughout the rest of the study). Ru@Pt/C and Pt/C samples were evaluated with a durability test procedure adapted from the DOE suggested protocol [53]. Briefly, the samples were cycled between 0.6 and 1.0 V vs. RHE at 125 mV s^−1^ in oxygen saturated electrolyte at room temperature. While the DOE protocol involves potential cycling in a membrane electrode assembly (MEA) setup at 80 °C, our stability investigation was conducted at room temperature in an RDE setup similar to other literature reports [21,72,73]. Catalyst performance metrics after specified numbers of durability cycles (1; 1000; 10,000; and 30,000 cycles) are summarized in Figure 5 and Table S2. The evolution of the mass activity is shown for Ru@Pt/C and Pt/C in Figure 5a. Overall, while both samples exhibit very good durability over the course of the stability test, Ru@Pt/C outperforms the Pt/C control. The first 1000 cycles had no clear effect on the mass activity of Pt/C, while the mass activity of Ru@Pt/C improved slightly, peaking at 0.54 A mg_Pt_^−1^. At 10,000 cycles, Ru@Pt/C returned to its initial mass activity, whereas Pt/C began to show signs of degradation. At the end of the stability test (cycle 30,000, samples denoted Ru@Pt/C-30k or Pt/C-30k) the mass activities of Ru@Pt/C-30k and Pt/C-30k are 0.43 and 0.28 A mg_Pt_^−1^, representing a 50% higher mass activity for Ru@Pt/C-30k in comparison to Pt/C-30k. Pt/C only retained 62% of its initial activity, while Ru@Pt/C retained 85% (see Figure 5d), far exceeding the DOE 2020 target for activity retention of >60% (although as mentioned previously, those targets refer to MEA testing at 80 °C) [53].

Changes in catalyst ECSA during stability testing (Figure 5b) show a loss in surface area for both catalysts. Similar Pt surface area losses were observed with CO stripping (Figure S7). Over 30,000 cycles, Ru@Pt/C loses only 20% of its ECSA (Figure 5d), with half of that loss (10%) occurring in the first 1000 cycles. In contrast, Pt/C barely loses any ECSA in the first 1000 cycles but subsequent degradation occurs at a faster rate than Ru@Pt/C, such that by the end of the test Pt/C has lost 35% of its initial ECSA. Further improvements in stability can likely be achieved though optimized annealing procedures and improved anchoring to the carbon support.

### 2.6. Material Stability

**Figure 6:**
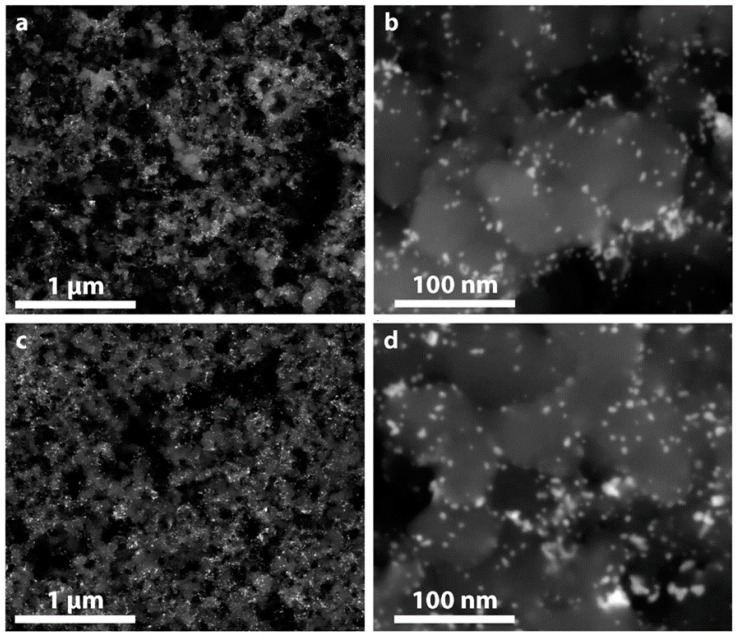
SEM images of Ru@Pt/C electrodes after (**a**,**b**) cycle 1 and (**c**,**d**) 30,000 stability cycles. The images use backscattered electrons for enhanced identification of the catalyst particles from the carbon support. The number of coalesced particles has not increased from cycle 1 to 30,000, which is consistent with the relatively small loss in ECSA.

To understand the physical processes underlying the stability trends observed electrochemically, scanning electron microscopy (SEM) images collected in backscatter mode of the electrodes before and after stability cycling are shown in Figure 6. Figure 6a,b shows the Ru@Pt/C electrode at cycle 1, after initial electrochemical conditioning/cleaning and ORR activity evaluation. The morphology of the electrode surface matches TEM observations shown in Figure 3, with metallic nanoparticles dispersed over a high surface area carbon support. Figure 6c,d shows SEM images of Ru@Pt/C electrodes after stability testing (additional images are shown in Figure S8 in the Supplementary Materials). There are no apparent differences in the particle size of the catalyst at cycle 30,000 compared to cycle 1, which is consistent with the relatively small loss in catalyst surface area (Figure 5b,d). Qualitatively, the number of coalesced particles does not appear to increase after 30,000 cycles (a quantitative analysis of particle size distributions before and after stability testing is presented in Figure S9 in the Supplementary Materials), indicating that the primary loss of catalyst surface area could be due to Pt dissolution or Ostwald ripening, although a more thorough investigation such as identical location imaging would be necessary to confirm this [15]. It is possible that the particles that are prone to coalescence (i.e., those in close proximity to other particles and/or poorly anchored to the carbon support) undergo coalescence during the initial electrochemical testing at cycle 1, leaving few particles with an easy pathway to coalesce during the subsequent stability cycling. Alternatively, the cycling conditions might not be aggressive enough to induce particle coalescence at an appreciable rate. The potential window is limited to 0.6–1.0 V vs. RHE during stability cycling to simulate operating conditions, while the catalyst is exposed to a wider window during conditioning (0.05–1.55 V) and nitrogen and oxygen CVs (0.05–1.1 V). Nonetheless, under the examined conditions, there is no noticeable change in the morphology of the Ru@Pt/C catalyst by SEM.

**Figure 7:**
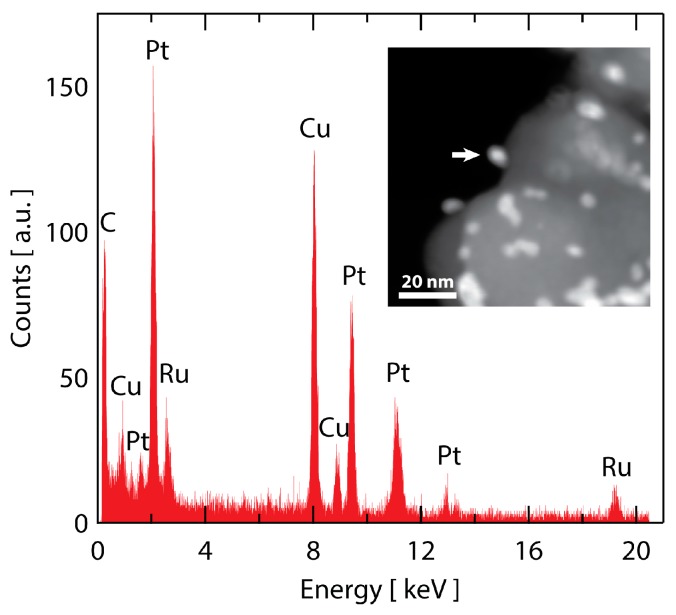
STEM analysis of Ru@Pt/C after 30,000 stability cycles. An EDS spectrum of a particle designated with the arrow in the inset dark-field STEM image. The spectrum shows a clear signal for Ru that remains in the particle after stability testing. As expected, there is also a strong signal from Pt (as well as Cu from the TEM grid and C from the support and TEM grid). The spectrum is representative of several particles that were analyzed.

To probe whether Ru is stable to dissolution from the particle core after 30,000 cycles, STEM-EDS was employed. A dark field STEM image of the Ru@Pt-30k catalyst is shown in the inset of Figure 7. The catalyst morphology is consistent with what was observed by SEM in Figure 6, with several metallic Ru@Pt/C particles with a portion showing evidence of coarsening. To probe the Ru content of the particles, spot selected EDS measurements on individual nanoparticles were collected with a typical spectrum shown in Figure 7 (location indicated by arrow in the inset STEM micrograph). The spectrum shows a clear presence of Ru, indicating the core-shell structure mitigates complete Ru dissolution even after 30,000 stability cycles under harsh O_2_ saturated 0.1 M HClO_4_ conditions. Additional 30,000 cycle EDS spectra are shown in Figure S10, confirming these observations.

Recently, it has been shown that unstable cores are likely to dissolve, even when protected by Pt shells, due to pinholes that form in the shell caused by room temperature thermal fluctuations [74]. However, for the Ru@Pt/C system studied here, we observe that the Pt shell offers reasonable protection for the otherwise unstable Ru core, both against extremely high oxidative potentials—1.55 V vs. RHE as demonstrated in Figure 3—and against long durations at moderately oxidizing potentials—1.0 V vs. RHE as demonstrated in Figure 7. As one would expect, previous work shows that the chance of pinhole formation in the Pt-shell decreases as the thickness of the shell increases [74]. Therefore, from a stability perspective, it may be desirable to have a Pt-shell thicker than one atomic layer. However, most metal-core Pt-shell systems have focused on single layer Pt-shell coverage to maximize Pt utilization and mass activity. The Ru@Pt/C system, however, maximizes its mass activity at thicker Pt layers (as demonstrated here and in a previous report [51]). This presents a nuanced benefit to the catalyst design approach outlined previously [52], namely that by using a core material that over-weakens the Pt-O bond (such as Ru or Rh, as opposed to Pd), a thicker Pt layer is needed to screen the core interaction which could have positive implications for stability.

## 3. Conclusions

In this study, we have demonstrated an improvement in ORR activity for liquid-phase synthesized Ru@Pt/C catalysts that achieve a Pt-based mass activity of 0.50 A mg_Pt_^−1^ at 0.9 V vs. RHE, which exceeds the state-of-the-art commercial Pt/C catalysts as well as the DOE 2020 target. Particle size, surface area, specific activity and mass activity was found to vary as a function of Pt content. An optimized mass activity was achieved at a Pt:Ru ratio of 1 and through the composition range investigated, all Ru@Pt/C samples exceeded mass activity values of 0.4 A mg_Pt_^−1^ at 0.9 V vs. RHE. The most active composition of Ru@Pt/C was found to be remarkably durable, maintaining 85% of its mass activity after 30,000 accelerated duribility cycles. Through STEM-EDS, we found that the otherwise unstable Ru core is protected to a large degree by the Pt shell, even up to potentials as high as 1.55 V vs. RHE. Finally, the high ORR activity and stability of Ru@Pt/C makes it an interesting system for future study, either by pursuing further improvements through optimization of particle size, shape, uniformity and dispersion, or through integration into MEAs for performance evaluation.

## Supplementary Materials

The following are available online at http://www.mdpi.com/2079-4991/8/1/38/s1, Experimental procedures and further analysis of the Ru@Pt/C catalyst are discussed in the Supplementary Materials.

## Appendix A

The Ru@Pt particles were synthesized using a procedure similar to that reported previously [52]. Briefly, ruthenium acetylacetonate and polyvinylpyrrolidone were dissolved in ethylene glycol and the solution was heated quickly to refluxing temperature to nucleate Ru nanoparticles. After cooling to room temperature, hexachloroplatinic acid was added and the solution was heated slowly such that the Pt coats the Ru nanoparticles, forming Ru-core Pt-shell (Ru@Pt) particles. The particles were dispersed on acid-treated carbon black (Vulcan XC-72) and washed by alternating centrifugation in acetone and sonication in a water/ethanol mixture. More details are provided in the Supplementary Materials.

There are three differences between the procedure used in this study and in our previous study [52], which are: the Pt:Ru precursor ratio, acid treatment of the carbon and removal of an intermediate temperature anneal. In this study, the Pt:Ru ratio was reduced from 2 to 1 in order to target smaller nanoparticles that had thinner Pt shells. The carbon support was treated in nitric acid at 80 °C in an attempt to improve catalyst dispersion by increasing the number of defect sites that could serve as anchors [75]. To mitigate particle coarsening, we did not employ the intermediate temperature anneal (300 °C in H_2_).

Electrochemical characterization was performed using a rotating disk electrode in 0.1 M HClO_4_ electrolyte, with an ink containing carbon-supported Ru@Pt/C nanoparticles drop-casted on polished glassy carbon disks to create a thin film. Prior to electrochemical testing, an electrochemical conditioning step was performed [55], involving 25 cycles at 500 mV s^−1^ between 0.05 and 1.55 V vs. RHE. Baseline CV and ORR measurements were carried out at 20 mV s^−1^ and electrode rotation of 1600 rpm in N_2_ and O_2_ saturated electrolytes, respectively. In all testing, a Pt wire was used as the counter electrode and a custom built reversible hydrogen electrode (RHE) was used as the reference electrode. More detailed discussions of experimental methods, including electrochemical testing, catalyst loading measurements and microscopy instrumentation are provided in the Supplementary Materials.
