# Supplementary material for: Engineering Ru@Pt Core-Shell Catalysts for Enhanced Electrochemical Oxygen Reduction Mass Activity and Stability
Source: Nanomaterials (Basel). 2018 Jan 12;8(1):38. doi: 10.3390/nano8010038 (PMC5791125; doi:10.3390/nano8010038)
Supplement: Supplementary file 1 [file nanomaterials-08-00038-s001.pdf]

Engineering Ru@Pt Core-Shell Catalysts for Enhanced Electrochemical Oxygen Reduction Mass Activity and Stability

Ariel Jackson [1], Alaina Strickler [1,2] Drew Higgins [1,2] and Thomas Francisco Jaramillo [1,2,*]

1. Ru@Pt Nanoparticle Synthesis

The particle and shell synthesis reactions are polyol reflux reactions carried out in a 100 mL 2-neck round bottom flask. A water-cooled condenser is fixed vertically above the central neck and is capped with a rubber septum pierced by a needle to equilibrate pressure. A thermocouple is inserted through a rubber septum in the side neck for monitoring the solution temperature. A hemispherical heating mantle connected to a computer controlled Omega temperature controller is used to heat the reaction (utilizing a second thermocouple, inserted between the mantle and flask, for feedback). In a typical synthesis, 39 ml of ethylene glycol is added to the flask, to which 40 mg of $Ru(acac)_3$ and 55 mg of PVP (MW = 55,000 g mol^{-1}) are added. The solution is stirred at 300 rpm and heated rapidly (~15 minutes) to refluxing temperature (~190°C). It is held under reflux for 3 hours, and then allowed to cool to room temperature, all with continuous stirring. After cooling, 1 ml (0.6 mL for $Ru@Pt_{0.6}$ and 2 mL for $Ru@Pt_2$) of 100 mM $H_2PtCl_6 \bullet 6H_2O$ in ethylene glycol is added to the Ru core solution. The solution is slowly heated (3 hour ramp to reflux) and held at reflux for 1.5 hours. After 1.5 hours, the heat is turned off and the mantle is lowered from the flask to facilitate rapid cooling to room temperature.

Vulcan XC-72 carbon support was treated in concentrated nitric acid at 80°C for 30 minutes, then rinsed, filtered and dried. 5.3 mg of the carbon support was placed in a 50 mL centrifuge tube. Typically, 2 mL of the glycol-nanoparticle solution and 20 mL of acetone was added to the tube and the mixture was mixed completely using a lab vortex. The nanoparticles were then centrifuged at 9,000 rpm (rcf ~9400) at 15°C for about 30 min, resulting in a clear supernatant. The supernatant was decanted and the particles were redispersed in a ~5 mL water/ethanol (ratio of ~1:1) mixture using agitation and sonication. This washing procedure was repeated for a total of 3 times. The samples were dried in glass vials in ambient atmosphere at 100°C overnight (0.3°C/min ramp). To form the catalyst ink, 1.8 mL water, 1.2 mL IPA, and 12 μL Nafion 117 (5% solution in alcohol and water, Aldrich) were added to the vial of dried powder. The mixture was sonicated using a mixture of bath sonication and horn ultrasonication (Qsonica Q125) until the ink was homogenous.

2. Electron Microscopy

To prepare samples for TEM imaging, samples were drop-cast onto ultra-thin or lacey carbon TEM grids (Ted Pella). To analyze samples after electrochemical testing, we used clean tweezers to scratch a small section of the (dry) glassy carbon electrode coated with catalyst, which was then transferred to a TEM grid by mechanical contact. TEM micrographs were obtained with an FEI Tecnai operated at 200 kV and an FEI Titan with spherical aberration objective lens correction at 300 kV. EDS spectra and elemental maps were taken in STEM mode using an EDAX SUTW (super ultra thin window) and analyzer with 0.3 srad EDS solid collection angle on the FEI Tecnai and Oxford SSD EDS detector on the FEI Titan. Ru-L and Pt-M peaks were used for elemental detection. Image and EDS processing was done using TIA (Tecnai Imaging and Analysis) and ImageJ software.

SEM was performed on an FEI Magellan 400 XHR Scanning Electron Microscope with FEG source. The catalyst was imaged directly on the glassy carbon electrode which was mounted to the SEM stage using silver paint (Ted Pella). A 5 kV beam at 50 μA was used and the stage was biased at 2000 V. The secondary electrons were captured with a through the lens detector (TLD) and the backscattered electrons were captured with a concentric ring backscattered electron detector (CBS).

2.1. Size Distribution

ImageJ software was used to obtain particle size statistics. The TEM images were processed into a binary black and white image and particle areas were determined using the software's automatic particle analysis feature. Appropriate filters were used to remove artifacts and overlapping particles, followed by manual removal of remaining artifacts. The particle area was converted into a diameter (assuming a circle of equal area) from at least 170 counted particles. Distributions were modeled with Gaussian functions to extract the mean and standard deviation of the core-shell particles.

The size distribution shown in Figure S1 for Ru@Pt_1 exhibited a higher fraction of uncoated Ru cores than our other Ru@Pt syntheses (0.6, 2.0), likely due to a longer heating time in the Pt coating step (24 vs. 3 hours). Improvements in the synthesis that would increase the number of Ru cores coated with Pt could enhance the performance in a few ways. Most directly, the uncoated Ru cores do not contribute to ORR current (they are removed by electrochemical conditioning and their ORR activity is orders of magnitude lower than Pt or Ru@Pt[S1]), so removing them at the point of synthesis would reduce the Ru mass loading without reducing the ORR current. Indirectly, more uncoated Ru cores indicates that more of the Pt went to fewer Ru@Pt particles, reducing the total number of available active particles and therefore availability of ORR reaction sites.

3. Measuring Pt Mass Loading

3.1. Mass Loading in Stability Tests

Precise measurements of the samples mass loadings are determined by ICP-MS, which requires the destruction of the sample by dissolution of the Pt in aqua regia. Because of the destructive nature of the measurement, it is impossible to obtain precise mass loadings before electrochemical testing of the catalysts for stability. While measurement of the Pt loading at the end of the stability test would be technically feasible, it would not be representative of the initial mass loading since we must allow for the possibility of Pt loss from the sample. The Pt loss could occur through a variety of mechanisms, including dissolution, Ru@Pt particle detachment, and carbon support detachment. Measuring the mass loading at the end of the stability test would give an artificially low Pt mass loading, and therefore a corresponding, artificially inflated mass activity. Since the technologically relevant number is the initial mass loading, i.e., how much Pt a fuel cell producer would be adding to the device at the time of manufacture, we obtained statistics on the reproducibility of our sample prep and loading procedures.

To obtain mass loading statistics, 10 μL aliquots of the catalyst ink were drop cast into 7 mL vials. The inks were mechanically mixed before drop casting by means of a lab vortex and bath sonicator. 1 mL of freshly prepared aqua regia was dropped into each vial and left overnight to dissolve the Pt. Then, the entire 1 mL of Pt/aqua regia solution was diluted in 39 mL of water. 4 independent drop cast samples were prepared from 3 different inks (12 samples total), and 2 ICP samples were prepared from each drop cast sample. From this data set we calculated the sample to sample variation that we would expect for electrochemical catalyst testing and obtained more accurate valuations for the Pt mass loading at the start of stability testing.

There are many processing steps required from synthesis of the particles to final RDE sample and although careful effort is taken to account for the mass loadings at each step, it is expected that there could be some catalyst loss at each step. From ICP analysis it was determined that the actual ink loading was ca. 66% of the nominal loading and the total coefficient of variation was determined to be 3% between 10 μL depositions. The average loading of the 4 depositions was used as the mass loading in the stability test shown in Figure 5 and the error bars are 2 standard deviations.

3.2. Mass Loading for Activity Measurements

Pt mass loading for Ru@$Pt_{0.6}$ and Ru@Pt_2 samples was determined using average drop cast loading as described for stability testing. For these samples, 3 independent drop cast samples were measured from each ink. To measure Pt loading in Ru@Pt samples shown in Figure 2, we used a

combination of average mass loading statistics (as described for stability testing) and measurements on individual samples. The average Pt mass was found to be consistent with the loadings measured on disks after electrochemical testing. To measure individual electrode samples, the individual disks were carefully removed from the Teflon RDE holder, after electrochemical testing, and deposited directly into a 7 mL glass vial. Care was taken not to scratch off any catalyst either during removal from the RDE shaft or when pushing the disk out of the holder directly into the glass vial. We added 1 mL of freshly prepared aqua regia to each vial. The vials were left uncapped overnight to dissolve the Pt. The full 1 mL of the aqua regia solution was diluted in 39 ml of Millipore water for ICP analysis.

4. Electrochemical Testing

The electrochemical characteristics and electrocatalytic performance of the Ru@Pt and Pt/C (46.6 wt. % on high surface area carbon, from TKK) catalysts were tested using a thin-film rotating disk electrode. A polished glassy carbon disk was set into a teflon holder and mounted to an RDE apparatus (Pine Research Instrumentation). The RDE was inverted so that the disk was pointed up. 10 μL of catalyst ink (equivalent to about 6 μg cm^{-2} for Ru@Pt and 6 or 14 μg cm^{-2} for Pt/C, see section on mass loading for more details) was drop-cast onto the disk and then the RDE was set to rotate at 700 rpm until the catalyst was dry, 20-40 minutes.[S2] The catalysts were tested in 0.1 M $HClO_4$ electrolyte and voltammograms were carried out at 20 mV s^{-1} at 1600 rpm. A Pt wire was used as the counter electrode, and a custom built reversible hydrogen electrode (RHE) was used as the reference electrode. The RHE was used instead of a commercial electrode such as $Hg/HgSO_4$ or Ag/AgCl, in order to prevent contamination of the electrolyte with sulfate or chloride anions, which have been shown to poison active sites on Pt-based catalysts.[S3,S4] Additionally, the use of a RHE eliminates the need for reference electrode calibration and electrode potential data post-correction. The series resistance of the cell was measured at 100 kHz and the IR losses were compensated at 85%. The typical compensated resistance was ~20 Ω.

Before electrochemical testing, the Ru@Pt catalysts were electrochemically conditioned up to 1.55 V based on the procedure developed in Ref. S5. Ru@Pt was cycled between 0.05–1.55 V at 500 mV s^{-1} for 25 cycles. The Pt/C catalysts were not subjected to the high potential electrochemical conditioning, since it was found that this procedure decreased the surface area and activity of the Pt/C catalyst. Following the conditioning, the Ru@Pt catalysts were electrochemically cleaned between 0.05–1.1 V at 500 mV s^{-1} for between 40 and 150 cycles, while the Pt/C catalysts were cleaned for either 0 or 40 cycles.

A baseline CV was run by cycling between 0.05–1.1 V at 20 mV s^{-1}. Before testing in oxygen, the electrolyte was removed, and fresh electrolyte was inserted into the cell. CVs were run from 0.05–1.1 V vs. RHE and the anodic going sweeps were extracted as linear sweep voltammograms. The oxygen purged sweeps are corrected for background current contributions by subtracting the baseline (nitrogen) anodic sweep from the oxygen sweep. The accuracy of the correction is verified by the fact that there is near zero current in the potential range of 1.0 to 1.1 V. In this range, there is not enough overpotential for oxygen reduction to occur, so the current should be zero. Without the background correction, current due to OH*/O* adsorption would be apparent.

To accurately compare the Ru@Pt and Pt/C catalysts, the specific activity (activity per real-Pt surface area) and mass activity (activity per Pt-mass) is required. First, the kinetic current is extracted by removing mass-transport effects from the voltammogram using equation 1.[S6] i_k is the kinetic current corrected for mass transport, i_d is the experimentally measured mass transport limited current, and i is the experimentally measured current. The kinetic current can then be normalized by the Pt-surface area or Pt-mass.

$$i_k = \frac{i_d i}{i_d - i} \quad (1)$$

The electrochemically active surface area (ECSA) is the real Pt surface area, accounting for all of the interfacial area between Pt and the electrolyte. The ECSA is calculated for each sample by integrating the double-layer corrected H_{UPD} charge. The average of the cathodic and anodic charge is

used and converted to a surface area using 210 μC cm_{Pt}^{-2}, which represents an estimate based on the average exposed facets on a bulk polycrystalline Pt surface.[S7] The area measured by the HUPD charge can be an underestimate if the adsorption is heavily suppressed resulting in major shifts of H_{UPD} onset.[S8] Although a small shift in the H_{UPD} region was observed, there was little difference between ECSA measured by CO stripping and H_{UPD} on the Ru@Pt catalysts. The CO stripping gave an ECSA that was 8% higher than when using H_{UPD}, which is in line with the 11% seen for the Pt/C control sample in Ref. S5. H_{UPD} was deemed suitable for surface area determination and was favored because CO stripping prevented the use of the RHE electrode (to avoid poisoning the Pt wire with CO). This would have made it difficult to simultaneously obtain accurate activity and surface area measurements on the same samples. However, care should be taken to verify that H_{UPD} remains a valid method of ECSA determination, especially as improvements to the Ru@Pt system are developed.

The process for CO stripping is as follows. CO was purged into the cell while the working electrode was held at 0.2 V vs. RHE for 1 min to chemisorb the CO to the catalyst surface. While keeping the potential at 0.2 V vs RHE, argon gas was purged into the cell to remove all non-adsorbed CO. After 20 minutes, the potential was swept anodic to 1.1 V vs RHE to oxidize the CO. Subsequent sweeps were performed to confirm full oxidation of CO and obtain a baseline scan. Both sweeps are shown in the plot. For these sets of experiments, a $Hg/HgSO_4$ reference electrode was used and calibrated to the RHE scale by performing a CV around 0 V vs. RHE while purging with H_2 (corresponding to ca. -0.720 V vs. $Hg/HgSO_4$). The samples were electrochemically cleaned prior to CO purging.

4.1. Adsorbate Potential Shifts

The shift of the H_{UPD} region is quantified by finding the zero-point of the slope of the baseline CV. This is shown in Figure S2. In the case of the Ru@Pt catalyst the zero-points for the anodic and cathodic sweeps overlap, each within 2 mV of 0.402 V. The Pt/C zero-points are separated, with the anodic sweep crossing zero between 32–45 mV (sample to sample variation) positive of the cathodic sweep. Comparing the H_{UPD} onsets between the Ru@Pt and Pt/C samples, the average (cathodic and anodic direction) Ru@Pt is shifted about 18–24 mV negative of Pt/C.

OH*/O* adsorption is more difficult to quantify since it does not have a peak in current, therefore we use only the desorption peak to quantify any shifts by finding the zero-point in the slope for the cathodic region. OH*/O* desorption is found to shift by 14–16 mV. Current from the carbon support could shift the apparent peak position from the true peak position, and it is possible that the most active sites (which contribute to the most ORR current) are not the most common (which is what the peak position would measure), however this analysis confirms that the adsorbate bond strength is trending in the desired direction compared to nanoparticulate Pt.

4.2. Comparison to Polyol-Pt

We also synthesized Pt nanoparticles using the same polyol synthesis that was used to synthesize the Ru nanoparticles/cores. The $Ru(acac)_3$ was replaced by $H_2PtCl_6 \bullet 6H_2O$. The polyol-Pt was then dispersed on Vulcan XC-72 carbon in the same way as the Ru@Pt particles. Electrochemical characterization of the polyol-Pt is shown in Figure S6. The poly-Pt has a larger particle size than TKK Pt/C and Ru@Pt leading to a more anodic OH*/O* desorption peak,[S6] however it has comparable specific activity while having much lower specific ECSA resulting in a lower mass activity. Ru@Pt exhibits an improved mass activity over polyol-Pt by a factor of 2.8 at 0.9 V vs. RHE.

5. Electrochemical Stability Testing

Prior to stability testing the catalysts were electrochemically conditioned (Ru@Pt only) and cleaned as described earlier. After cleaning, the electrolyte was replaced and N_2 and O_2 CVs for cycle 1 were collected at 1600 rpm. The catalyst was then cycled between 0.6–1.0 V at 125 mV s^{-1} at 0 rpm. After cycling, the cycled electrolyte was replaced with fresh electrolyte, the catalyst was cleaned

(0.05–1.1 V at 500 mV s^{-1}), and N_2 and O_2 CVs were obtained. We have found that these first two steps are crucial to measuring optimal activity. Typically tens to hundreds of cleaning cycles are performed, usually more cycles are required the longer the catalyst has been sitting in the electrolyte.

Figure S7 shows two SEM (backscattered electron) images at the same magnification after 1 and 30,000 stability cycles. Image analysis is used to count the particles, shown outlined in yellow, and generate histograms of particle size. The histograms are fit using log-normal distributions. The degree of similarity between the histograms and fitted functions of cycle 1 data and cycle 30,000 data further confirms the stability of the Ru@Pt catalyst under accelerated stability testing. Care was taken to optimize the image background subtraction and particle analysis parameters, however the process is not perfect. Particles that do not have enough contrast from the carbon support were not counted, and not every particle was outlined precisely. However, we believe that the very high (>1,000) number of particles counted allows us to draw conclusions despite the imperfect image analysis.

The stability of Ru@Pt is further demonstrated by CO stripping experiments shown in Figure S8. A monolayer of CO is adsorbed to Ru@Pt at 0.2 V vs RHE and then oxidized by sweeping anodically to 1.1 V vs RHE. The Ru@Pt-30k sample is shown in dark red and a sister sample of Ru@Pt (which has been electrochemically conditioned, but not subjected to stability cycling) is shown in light red. The CO stripping peak is the large peak centered around 0.8 V vs RHE. The Ru@Pt-30k sample has a slightly smaller CO stripping peak than the uncycled Ru@Pt sample (0.33 mC vs 0.39 mC), which is consistent with the smaller HUPD area visible in the CV and loss of ECSA observed in the stability test. There is little observed change in location of the CO stripping peak after 30,000 cycles, which is a further testament to the stability of Ru@Pt. A small shoulder in the peak around 0.75 V is present in the uncycled Ru@Pt that is not prominent in Ru@Pt-30k. This small feature could indicate a change in the adsorbate binding properties after 30,000 cycles, however the feature shape was found to be highly dependent on sample age, exposure to ambient atmosphere, and extent of electrochemical cleaning. This is consistent with other published literature on CO stripping on Pt catalysts.[S9] Thus, further study would be necessary to understand the nature of this particular feature. To obtain reproducible scans, each sample shown in Figure S8 was electrochemically cleaned (0.05–1.1 V vs RHE at 500 mV s−1 for ~150 cycles) prior to CO stripping.

Further confirmation of the Ru@Pt-30k catalyst morphology is shown in Figure S9. Images were taken from 3 different spots on the electrode at low magnification. Images of Pt/C-30k are included for comparison. In the Pt/C-30k sample, there are clear examples of large Pt agglomerates spanning >100 nm. These agglomerates were also observed in the as received Pt/C sample, so they are not formed due to the stability cycling. However, they are included here as a visual example of particle coalescence at this scale. It is clear that the Ru@Pt catalyst remains well dispersed without major particle growth or coalescence after the 30,000 cycles stability test.

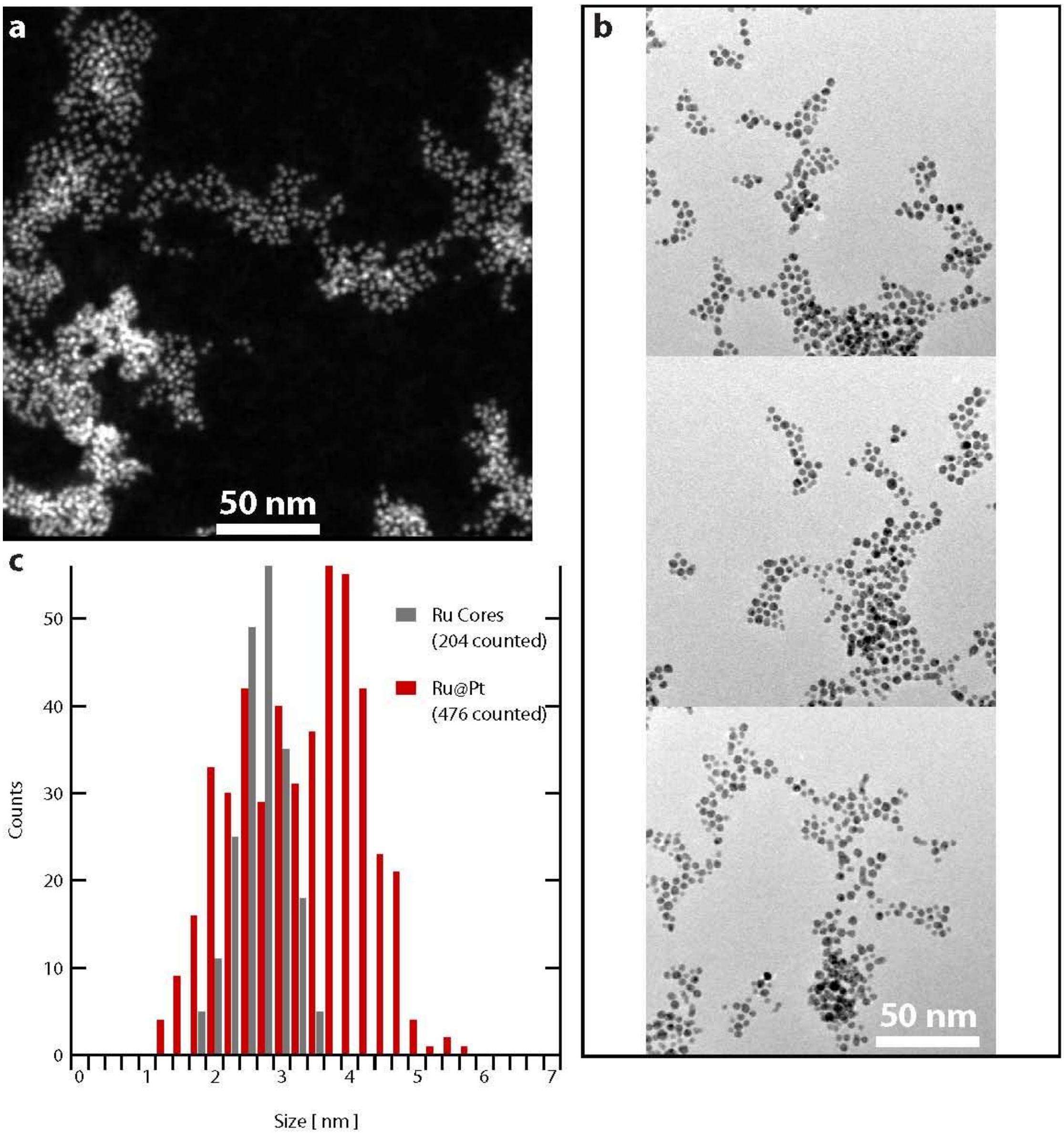

Figure S1. Particle Size Analysis of Ru@Pt. (a) DF-STEM image of Pt free Ru nanoparticles (cores). (b) TEM images of Ru@Pt particles (not supported on carbon). (c) Histogram of particle sizes for Ru cores (gray) and Ru@Pt (red) computed from the images in a and b. The particle counting parameters were tuned to exclude overlapping particles, while minimizing exclusion of true particles. 3 images of Ru@Pt (shown in b) were used to obtain sufficient particle counts. Gaussians were used to model the peaks and the mean and standard deviations of Ru cores and Ru@Pt particle diameters are 2.68 ± 0.36 and 3.95 ± 0.48 nm respectively.

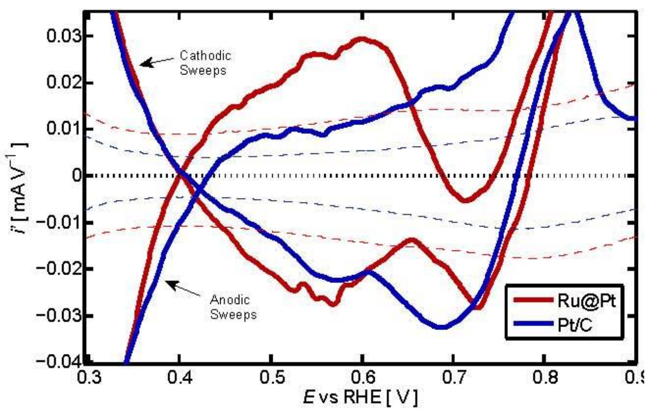

Figure S2. Differential of the Baseline CV. The raw CV shapes are presented in the dashed lines.

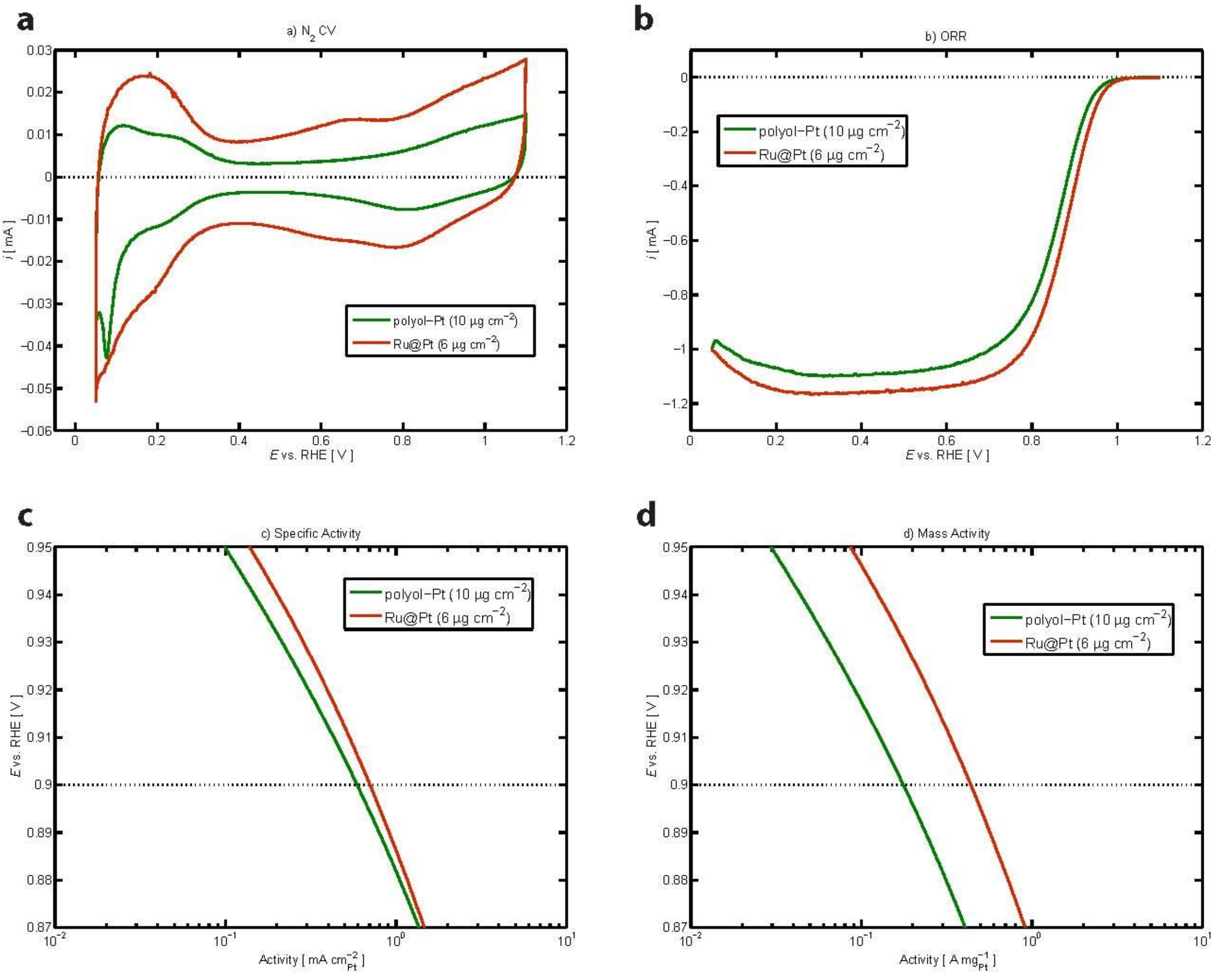

Figure S3. Electrochemical properties of Ru@Pt and polyol-Pt. All tests were performed at 20mV s^{-1} in 0.1 M $HClO_4$ at 1600 rpm using a reversible hydrogen electrode. (a) Cyclic voltammograms in N_2 saturated electrolyte. (b) Anodic direction linear sweep voltammograms in O_2 saturated electrolyte. The sweeps are corrected for background current by subtracting the N_2 anodic voltammogram. (c,d) Specific and mass activity of the catalysts.

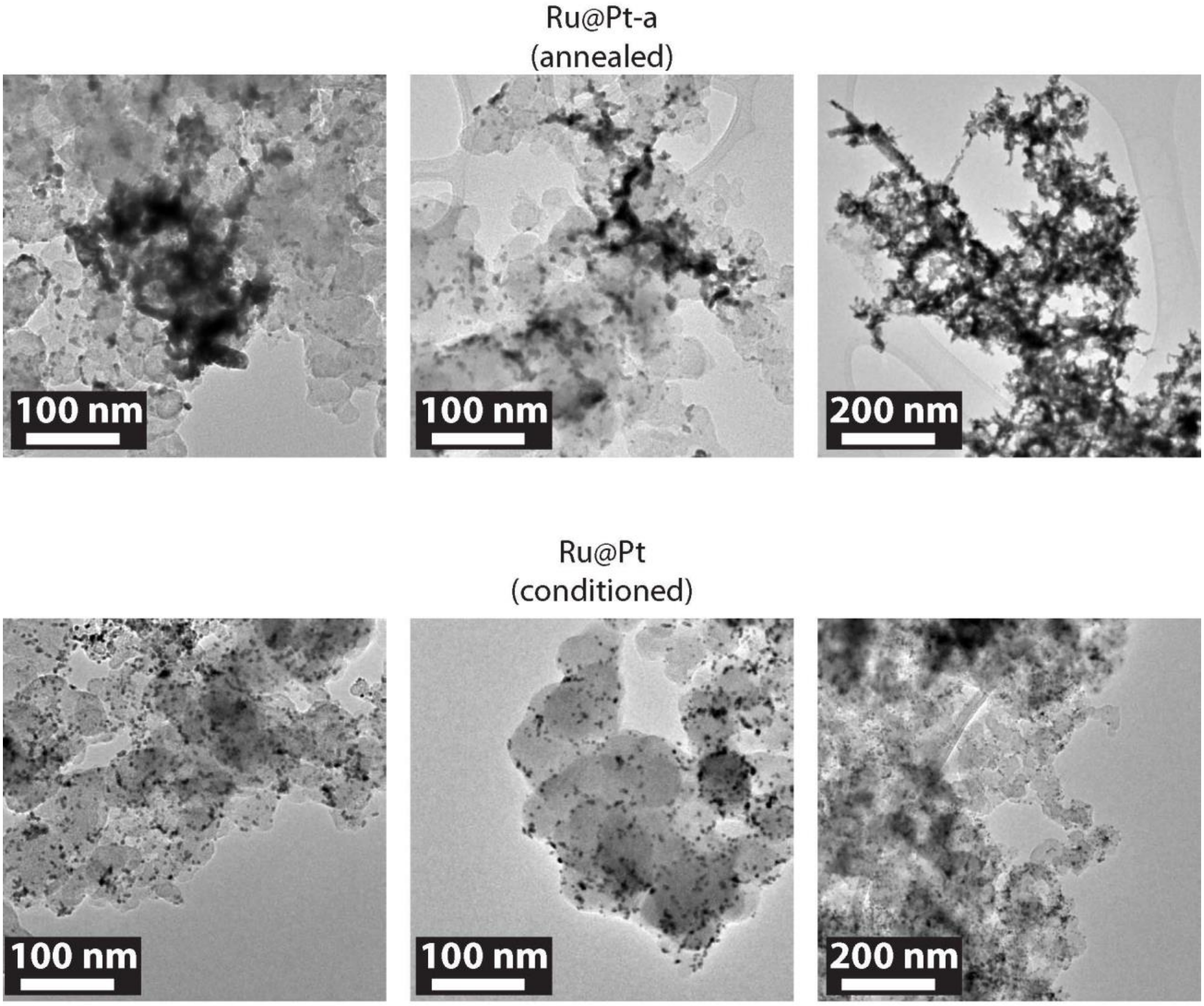

Figure S4. Comparison of particle coarsening among Ru@Pt catalysts. Images for catalysts that have and have not been annealed are shown. The annealing treatment involved heating to 300°C for 2 hours in hydrogen. The catalysts that have not been annealed have instead undergone electrochemical conditioning by cycling between 0.05 and 1.55 V, as well as further ORR testing between 0.05 and 1.1 V. The images of the annealed catalysts show examples of very large (>100 nm) agglomerates which were not detected in the conditioned catalysts.

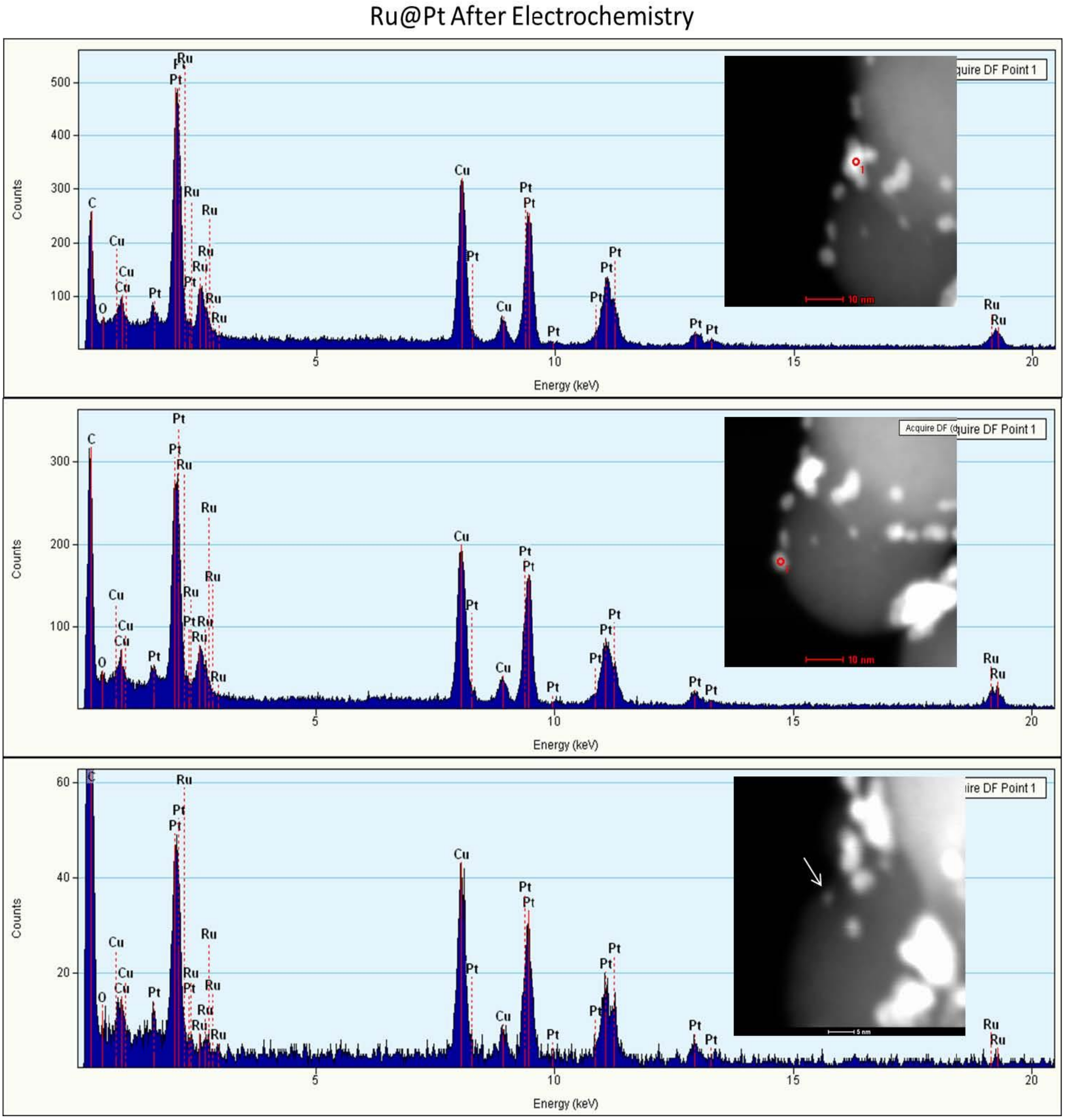

Figure S5. Additional EDS spectra of Ru@Pt particles after electrochemistry (consisting of conditioning up to 1.55 V, cleaning, and cycling in N_2 and O_2 up to 1.1 V). (1 of 2)

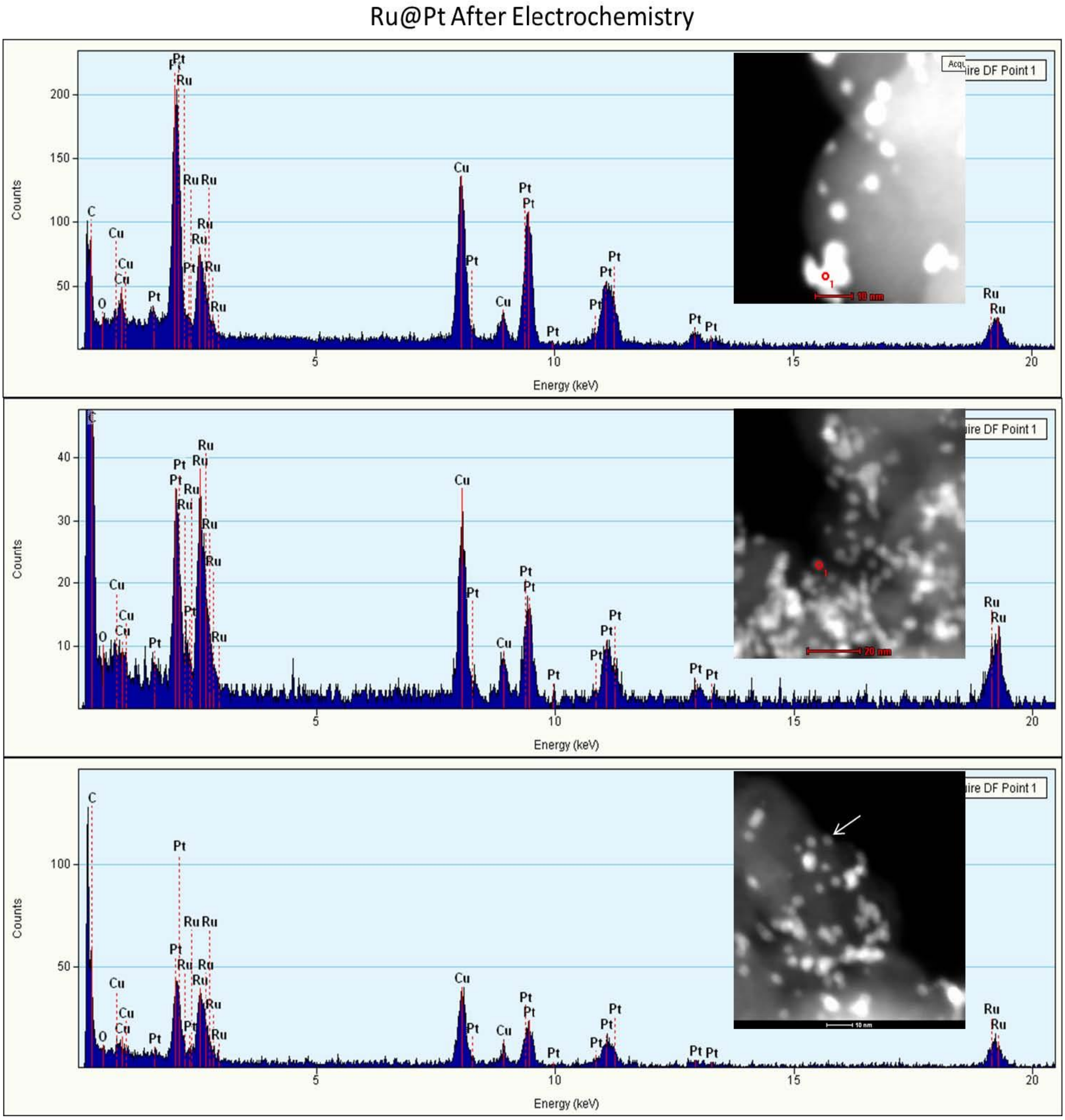

Figure S6. Additional EDS spectra of Ru@Pt particles after electrochemistry. (consisting of conditioning up to 1.55 V, cleaning, and cycling in N_2 and O_2 up to 1.1 V.) (2 of 2)

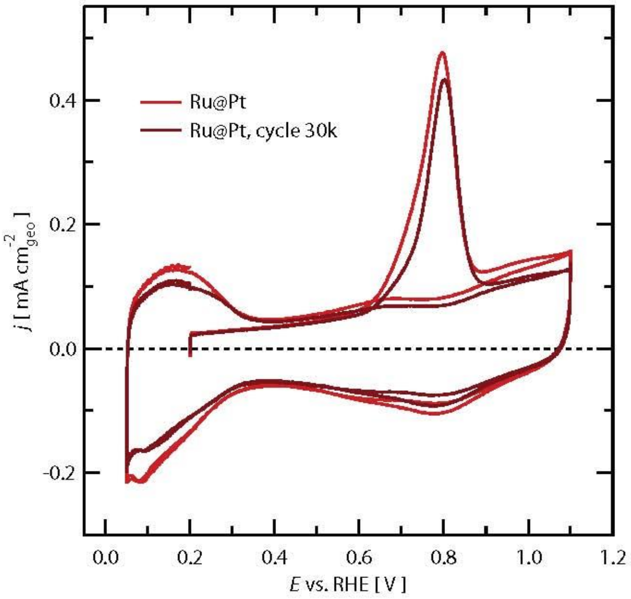

Figure S7. CO stripping on Ru@Pt before and after stability testing.

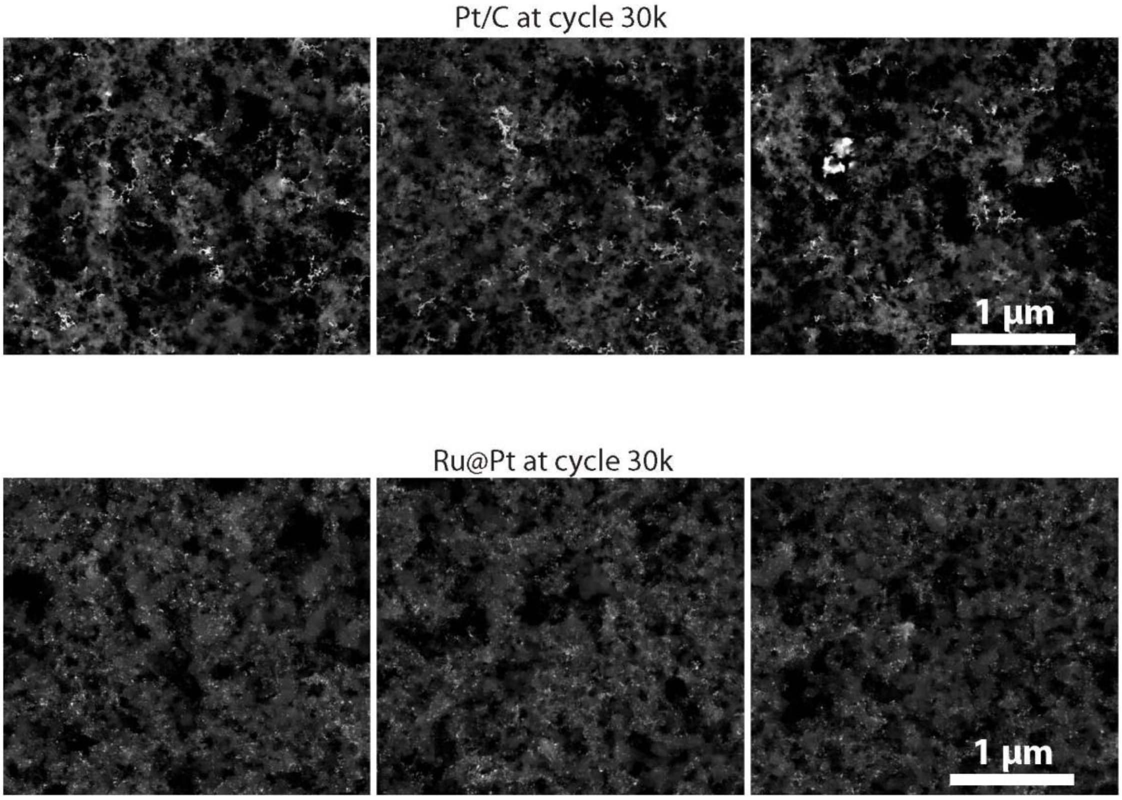

Figure S8. Additional SEM images of Ru@Pt and Pt/C at 30,000 stability cycles.

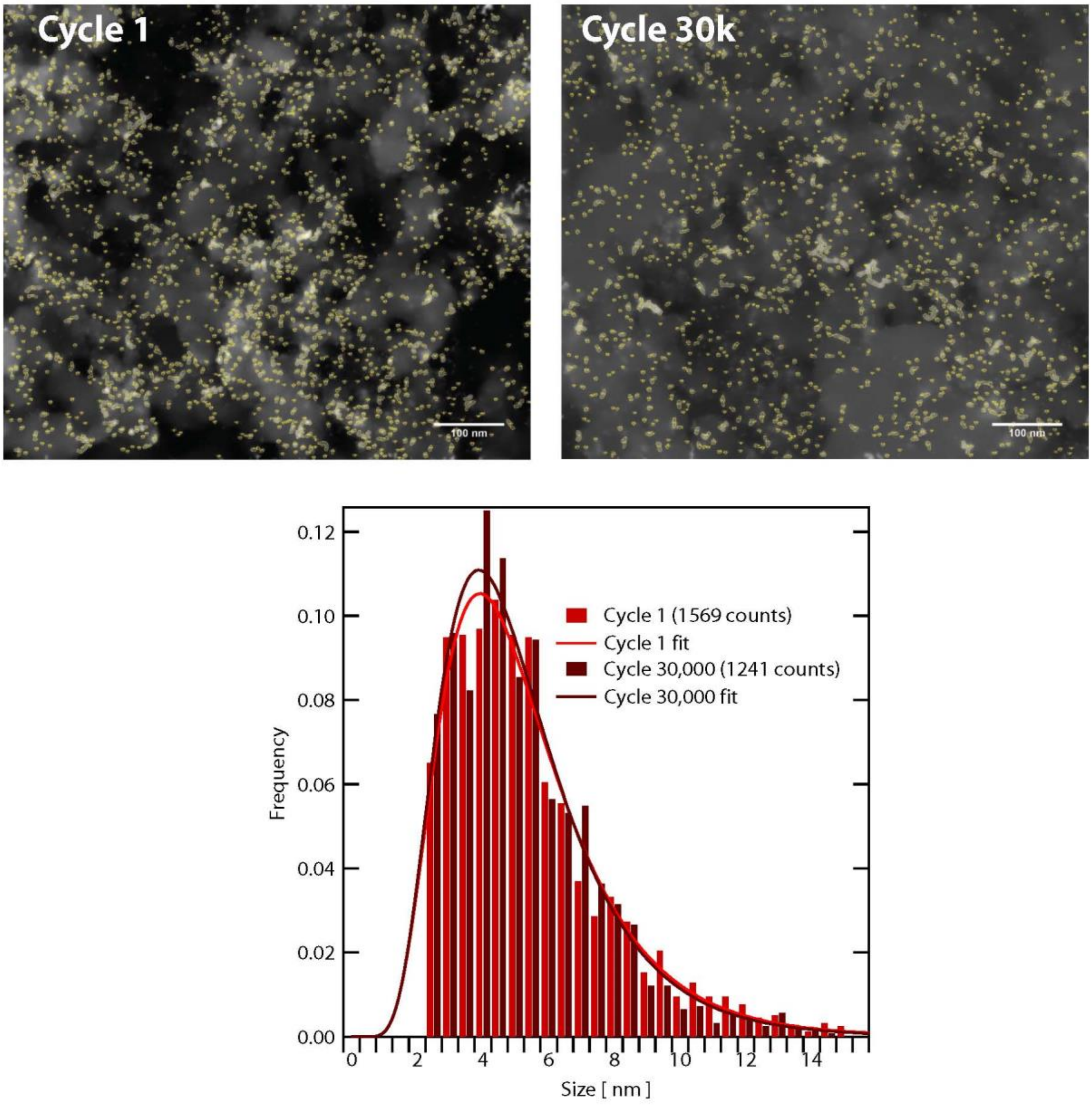

Figure S9. SEM images at the same magnification after 1 and 30,000 stability cycles. Image analysis is used to count particles (outlined in yellow) and generate particle size histograms. The histograms are clipped below a particle size of 2 nm, where it becomes difficult to distinguish image noise from small particles at this magnification and image resolution (Horizontal field width of 746 nm in 2048 pixels). The histograms are fit with log-normal distributions. As can be seen by both the histograms and the fits, there is very little change in particle size after 30,000 stability cycles.

Ru@Pt, Cycle 30,000

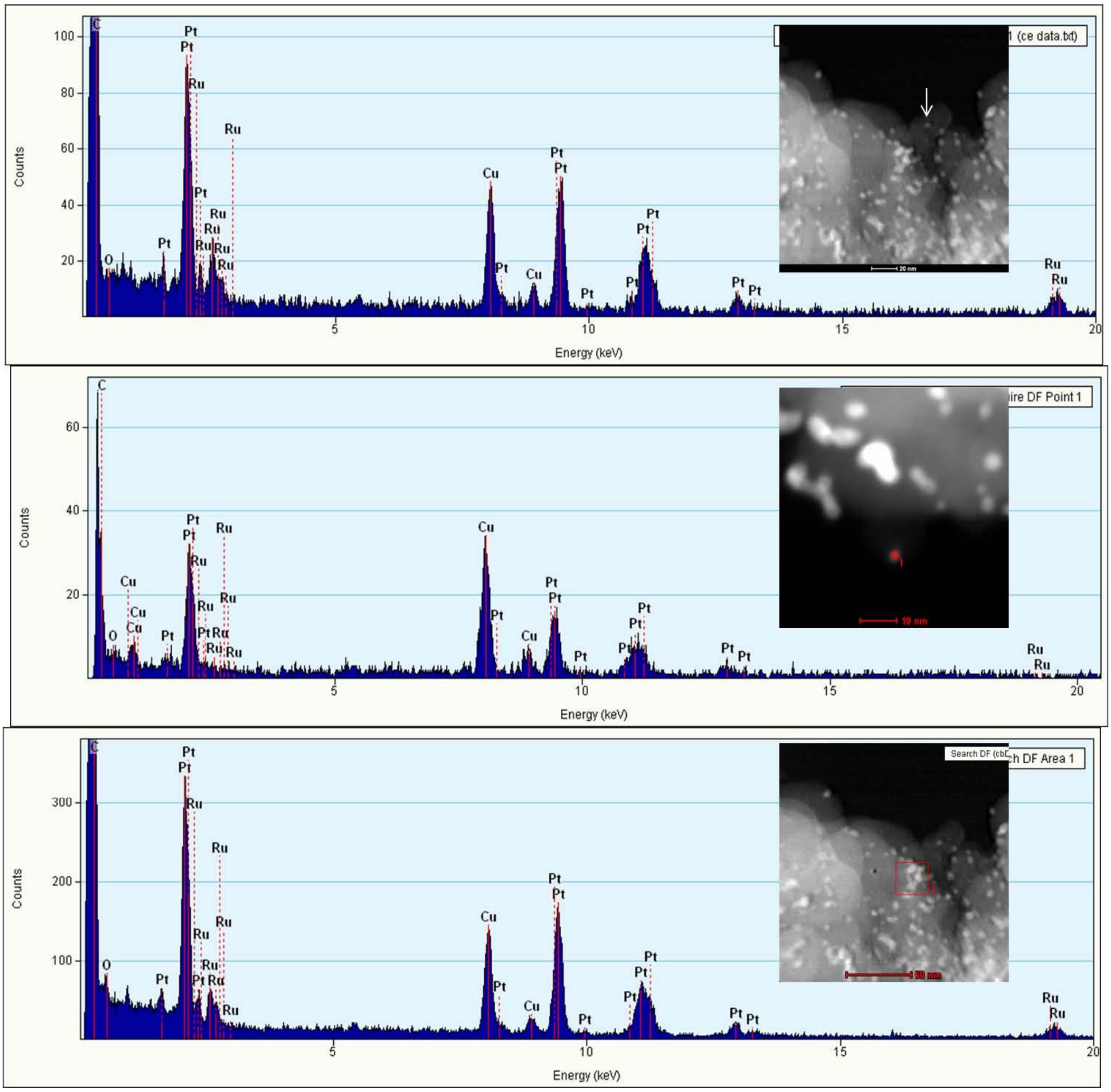

Figure S10. Additional EDS spectra of Ru@Pt after 30,000 stability cycles

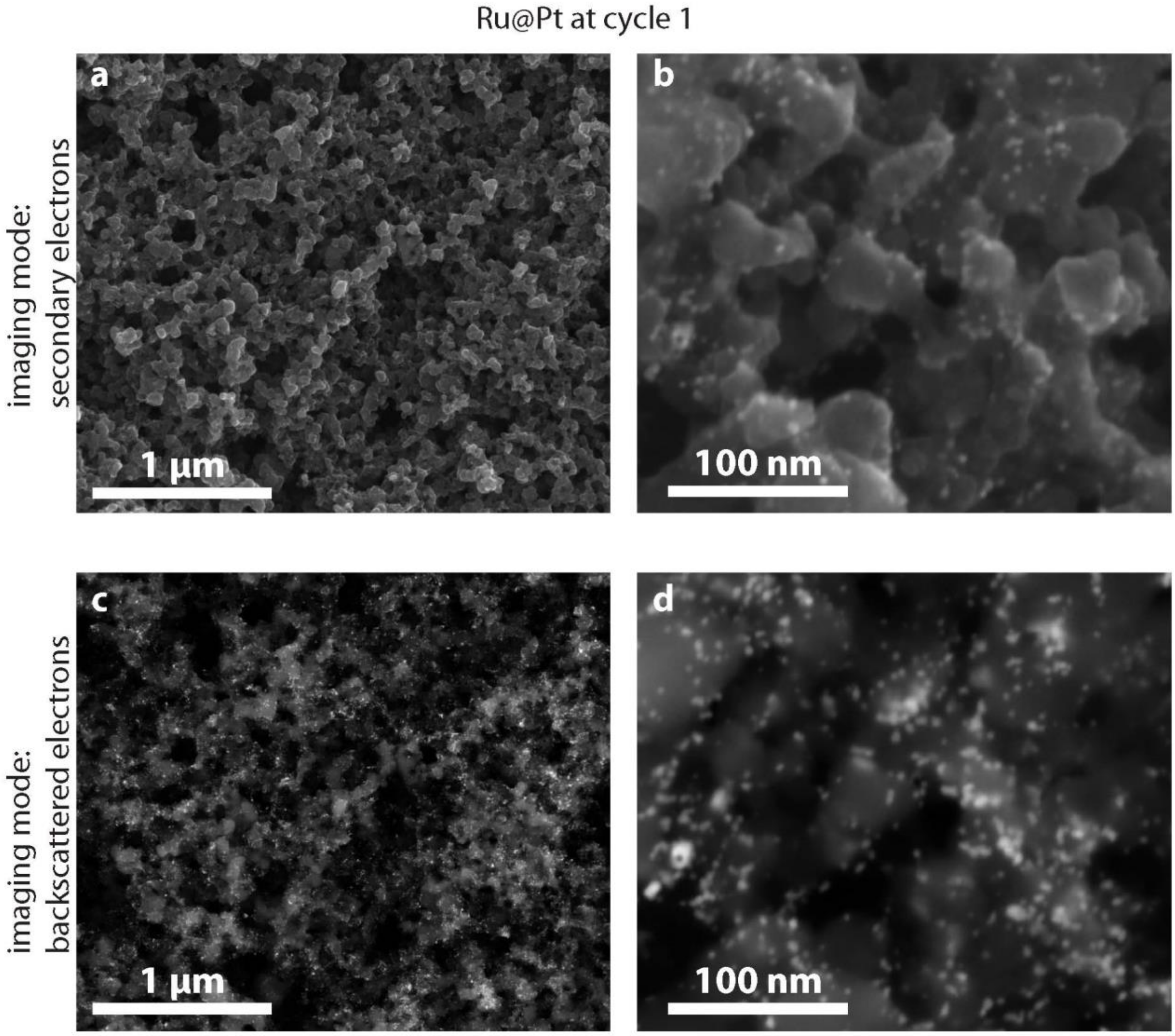

Figure S11. SEM images of Ru@Pt catalyst at cycle 1. Two imaging modes are used; (a,b) secondary electrons are the most common imaging source and provide visual information about the surface topography, (c,d) backscattered electrons are more sensitive to atomic number and allow for enhanced identification of the metal catalyst particles from the carbon support. The images are collected simultaneously so that the low magnification images (a,c) and high magnification images (b,d) represent the same exact field of view.

Table S1. Comparison of Ru@Pt/C catalysts with different Pt:Ru ratios. The particle size is measured by TEM. The specific and mass activity values are measured at 0.9 V vs. RHE.

Pt : Ru Molar Ratio	Particle Size [nm]	Specific ECSA [$m^2\ g_{Pt}^{-1}$]	Specific Activity [$mA\ cm_{Pt}^{-2}$]	Mass Activity [$A\ mg_{Pt}^{-1}$]
0	2.7 ± 0.4	-	-	-
0.6	3.0 ± 0.7	82 ± 2	0.54 ± 0.01	0.45 ± 0.01
1	4.0 ± 0.5	71 ± 2	0.70 ± 0.01	0.50 ± 0.02
2	5.3 ± 0.4	48 ± 1	0.85 ± 0.01	0.41 ± 0.01

Table S2. Results of stability testing. The specific activity and mass activity values are taken at 0.9 V vs. RHE. The stability test involves cycling in O_2 saturated 0.1 M $HClO_4$ from 0.6–1.1 V vs. RHE at 125 mV s^{-1} at room temperature.

Cycle	Specific ECSA	Specific Activity	Mass Activity
	[$m^2\ g_{Pt}^{-1}$]	[mA cm_{Pt}^{-2}]	[A mg_{Pt}^{-1}]
		Ru@Pt/C	
1	71 ± 5	0.70	0.50 ± 0.03
1,000	65 ± 4	0.83	0.54 ± 0.03
10,000	60 ± 4	0.82	0.50 ± 0.03
30,000	57 ± 4	0.75	0.43 ± 0.03
		Pt/C	
1	72 ± 5	0.64	0.46 ± 0.03
1,000	70 ± 4	0.66	0.46 ± 0.03
10,000	56 ± 4	0.73	0.41 ± 0.03
30,000	48 ± 3	0.59	0.28 ± 0.02

References

(S1) Zaikovskii, V. I.; Nagabhushana, K. S.; Kriventsov, V. V.; Loponov, K. N.; Cherepanova, S. V.; Kvon, R. I.; Bonnemann, H.; Kochubey, D. I.; Savinova, E. R. *The Journal of Physical Chemistry. B* 2006, 110, 6881–90.

(S2) Garsany, Y.; Singer, I. L.; Swider-Lyons, K. E. *Journal of Electroanalytical Chemistry* 2011, 662, 396–406.

(S3) Strmcnik, D.; Escudero-Escribano, M.; Kodama, K.; Stamenkovic, V. R.; Cuesta, A.; Markovic, N. M. *Nature Chemistry* 2010, 2, 880–5.

(S4) Schmidt, T.; Paulus, U.; Gasteiger, H.; Behm, R. *Journal of Electroanalytical Chemistry* 2001, 508, 41–47.

(S5) Jackson, A.; Viswanathan, V.; Forman, A. J.; Nørskov, J. K.; Jaramillo, T. F. *ECS Transactions* 2013, 58, 929–936.

(S6) Gasteiger, H. A.; Kocha, S. S.; Sompalli, B.; Wagner, F. T. *Applied Catalysis B: Environmental* 2005, 56, 9–35.

(S7) Green, C. L.; Kucernak, A. *The Journal of Physical Chemistry B* 2002, 106, 1036–1047.

(S8) van der Vliet, D. F.; Wang, C.; Li, D.; Paulikas, A. P.; Greeley, J.; Rankin, R. B.; Strmcnik, D.; Tripkovic, D.; Markovic, N. M.; Stamenkovic, V. R. *Angewandte Chemie* 2012, 124, 3193–3196.

(S9) Rudi, S.; Cui, C.; Gan, L.; Strasser, P. *Electrocatalysis* 2014, 5, 408–418.
